# Effects of Ferulic Acid on Respiratory Metabolism, Oxidative Lesions, and Apoptotic Parameters in Gills and Red Blood Cells of Carp (*Cyprinus carpio* Var. Jian) Response to Copper

**DOI:** 10.3390/antiox13030314

**Published:** 2024-03-04

**Authors:** Huatao Li, Haijing Liu, Siyue Wu, Chengyan Ai, Qi Yang, Jingting Jia, Xiao Xu, Min Wu, Jun Jiang

**Affiliations:** 1 Key Laboratory of Sichuan Province for Conservation and Utilization of Fishes Resources in the Upper Reaches of the Yangtze River, Neijiang Normal University, Neijiang 641100, China; 2 College of Life Sciences, Neijiang Normal University, Neijiang 641100, China; 20191642024@stu.njtc.edu.cn (H.L.); 20201642037@stu.njtc.edu.cn (S.W.); 20201643074@stu.njtc.edu.cn (C.A.); 20201643021@stu.njtc.edu.cn (Q.Y.); 20201642017@stu.njtc.edu.cn (J.J.); 20211641005@stu.njtc.edu.cn (X.X.); 3 Archives, Neijiang Normal University, Neijiang 641100, China; 40001246@njtc.edu.cn; 4 College of Animal Science and Technology, Sichuan Agricultural University, Chengdu 611130, China; jjun@sicau.edu.cn

**Keywords:** Cu, ferulic acid, respiratory metabolism, oxidative lesion, apoptosis, gills, red blood cells

## Abstract

In sustainable aquaculture systems, copper sulfate (CuSO_4_) is widely applied as a disinfectant to control parasitic infections and algal growth. However, aquatic organisms may suffer from exposure to excessive concentrations of Cu. Elevated Cu concentrations could activate damage to the respiratory functions of aquatic animals. Thus, this study explored the effects exerted by ferulic acid (FA) on respiratory metabolism, oxidation-related lesions, and the apoptosis parameters of the gills and red blood cells in copper sulfate pentahydrate (CuSO_4_·5H_2_O)-treated carp (*Cyprinus carpio* var. Jian). When the 30-day feeding experiment was completed, the carp were exposed to 12.5 μM of Cu for 4 days. The results indicated that the Cu decreased the oxygen consumption and ammonia excretion rates in the carp, reduced the metabolic- and antioxidant-related enzyme activities and glutathione levels in the carp, and enhanced the caspase activities and reactive oxygen species and malondialdehyde levels in the gills of the carp. Moreover, in addition to the changes in the above parameters, the Cu decreased the cell numbers and hemoglobin concentrations and increased the phosphatidylserine exposure and cytochrome c levels in the red blood cells of the carp. These results demonstrate that Cu is capable of decreasing respiratory metabolism and increasing oxidation-related lesions and apoptosis inside the gills and red blood cells of the fish. However, dietary FA quenched the Cu-induced apoptosis and oxidative lesions by reversing the same biomarker indicators, thereby suppressing the Cu-induced decrease in respiratory metabolism. Thus, FA can be used as a suppressor of Cu stress in fish.

## 1. Introduction

Copper (Cu), as a micronutrient, plays an essential role in animal growth and development [1]. The Cu concentration recommended by the U.S. EPA (1984) for the protection of aquatic organisms is 20 μg/L [2]. However, Cu is considered to be one of the most important pollutants both along marine coasts and in fishponds. The reason for this is that Cu is widely used in industries and is present in most wastewaters discharged into the sea [3,4]. In aquaculture systems, copper sulfate (CuSO_4_) is regularly used to control algal growth, parasitic infections, and saprolegniasis in concentrations varying from 0.5 to 2.0 mg/L [5,6]. Thus, aquatic organisms may suffer from exposure to excessive concentrations of Cu.

Gills are vital organs for respiration that control nitrogen excretion and gas exchange in fish [7]. Research has shown the primary accretion of Cu in the gills of fish [8]. Cu stimulates the overproduction of reactive oxygen species (ROS) [9], resulting in oxidative lesions in fish gill cells [10,11]. Moreover, Cu induces apoptosis via the extrinsic caspase-8 pathway and intrinsic caspase-9 pathway in fish gills [12]. As hemoglobin (Hb) is the most important oxygen carrier, red blood cells play a fundamental role in the respiratory activity in vertebrates [13]. It has been reported that fish can absorb Cu through their gills, body surfaces, and digestive tracts [14] and significantly increase the content of Cu in the blood [5,15]. Guo et al. (2017) report that Cu led to oxidative stress and apoptosis in the hemocytes of white shrimp [16]. Furthermore, Cu exposure reduces respiratory metabolism in common carp [17]. However, not much information on how Cu affects red blood cell oxidative lesions, apoptosis, and metabolism in fish can be found. Enzyme-related antioxidants, including glutathione peroxidase (GPx), catalase (CAT), and superoxide dismutase (SOD), are capable of scavenging ROS at a rapid speed and prevent oxidative stress in organisms [18]. Nonetheless, it has been reported that CuSO_4_ has the ability to change the defensive mechanisms of antioxidants [19,20] and leads to decreases in the immunity and performance of fish [21,22,23,24,25]. The factors mentioned above require us to broaden our knowledge regarding the protection of fish gills and red blood cells from oxidative lesions and apoptosis caused by CuSO_4_.

It has been reported that the phenolic compounds from terrestrial plants possess strong antioxidant activities [26]. Among them, ferulic acid (FA) is a common active ingredient in plants such as *Ferula sinkiangensis*, *Angelica sinensis*, *Ligusticum sinense*, and *Actaea cimicifuga*. Dietary FA supplementation is reported to increase growth performance and antioxidant capacity [27] and improve oxidized-fish-oil-induced oxidative stress in *Oreochromis niloticus* [28]. FA can be absorbed effectually through the intestinal mucosa inside mice [29] and easily move into the bloodstream [30]. Furthermore, the ability of FA to lower the ROS levels in the aortas of rats has also been reported [31]. Therefore, a reasonable hypothesis is that FA can mitigate the oxidative lesions and apoptosis caused by CuSO_4_ in the gills and red blood cells of fish in aquaculture ponds. However, there is a lack of information on the way in which FA affects fish gills and red blood cells after CuSO_4_ treatment.

This study investigated the respiratory metabolism, oxidative lesions, and apoptosis parameters in the gills and red blood cells of carp (*Cyprinus carpio* var. Jian) with the aim of evaluating the effects of FA on the actions of the gills and red blood cells in the fish under CuSO_4_ treatment. The research outcome underpins the application of FA as a natural Cu stress suppressor in fish.

## 2. Materials and Methods

### 2.1. Chemicals

Xi’an Baoyifeng Biotechnology Co., Ltd. (Shanxi, China), provided the FA (≥98%). Analytical-quality copper sulfate pentahydrate (CuSO_4_•5H_2_O) was supplied by the Shanghai Chemical Reagent Factory (Shanghai, China). Heparin sodium (≥99%) was provided by Sigma-Aldrich Co., LLC (St. Louis, MO, USA). Physiological carp saline (PCS) was prepared in the authors’ lab and contained (in mmol L^−1^) 1.43 KCl, 141.10 NaCl, 2.64 NaHCO_3_, 6.16 glucose, and 0.99 CaCl_2_, which were adjusted to achieve a pH of 7.9. The rest of the chemicals were, overall, of analytical grade.

### 2.2. Animal Experiment

#### 2.2.1. Experiment Diets

In this study, 5 experiment diets, including 1 basal diet and 4 FA diets, were formulated and produced following a previous procedure [32]. The basic diet involved 5.57% crude lipids and 33.89% crude proteins. FA was added to the basic diet to provide control, 0.10, 0.20, 0.30, and 0.40 g of FA per kg^−1^ of diet. The formulation of the feed pellets was adjusted by decreasing the cellulose content to balance the incorporation of FA. Each feed pellet formulation was tailored for a distinct treatment and underwent a drying process at 50 °C for 24 h in an oven. Subsequently, these feed pellets were meticulously packed into airtight sample bags and preserved in a controlled environment at −20 °C until required for experimental use. Proximate analyses of the diets were carried out on the basis of the methods of the AOAC (Association of Official Analytical Chemists) [33,34], looking at the dry matter, crude proteins and lipids, and ash. Table 1 presents the diet formulations.

#### 2.2.2. Feeding Trial

A fish farm (Neijiang, China) offered juvenile Jian carp, which were acclimated to the experiment environment according to Li et al. (2019) [32]. The standards, following the description of Li et al. (2020) [35], were met regarding water indices of dissolved oxygen, pH, ammonium–nitrogen, and nitrite. The water quality parameters were determined using the technique of Li et al. (2020) [35]. Following a 15-day period of acclimation to the laboratory settings, 400 fish with an average body weight of 9.68 ± 0.05 g were randomly assigned to 5 groups, each consisting of 4 replicate aquariums. The corresponding aquariums measured 55 cm by 32 cm by 40 cm and held 20 fish. The basic diet and FA diets containing 0.1, 0.2, 0.3, and 0.4 g of FA per kg^−1^ of diet were fed to the 5 groups (the control, 0.1, 0.2, 0.3, and 0.4 FA groups) for 30 days. All the fish were fed six times a day and subjected to strict inspections to ensure that they were sated and did not overfeed within the 30 days. During the study, any feed particles that remained uneaten were gathered, subjected to a drying process, and subsequently weighed. These data were crucial for accurately adjusting the calculations of the feed consumed. Upon completion of the feeding trial, the researchers meticulously recorded the number and weights of the fish in each aquarium. They utilized both the initial and final counts, along with the initial body weight (IBW), final body weight (FBW), and amount of feed consumed, to determine several key parameters: the survival rate (SR), weight gain (WG), specific growth rate (SGR), feed intake (FI), and feed efficiency (FE).

SR (%) = 100 × (final number/initial number of fish) [36]

WG (g fish^−1^) = final weight (g fish^−1^) − initial weight (g fish^−1^) [33]

SGR (% day^−1^) = 100 × (ln final weight − ln initial weight)/experimental duration (day) [37]

FI (g fish^−1^) = feed consumed (g)/final number of fish [38]

FE (%) = 100 × WG (g fish^−1^)/FI (g fish^−1^) [39]

#### 2.2.3. CuSO_4_ Exposure

Previous studies in our laboratory showed that CuSO_4_ at a concentration of 0.8 mg Cu per L^−1^ water induced the minimum FI value, which failed to cause death among the fish [40]. Thus, 0.8 mg L^−1^ was selected as the proper concentration of Cu for the current experiment. When the feeding experiment was completed, 30 fish from the control, 0.1, 0.2, 0.3, and 0.4 FA groups were exposed to 3.125 mg CuSO_4_·5H_2_O per L^−1^ water (0.8 mg L^−1^ or 12.5 μM Cu) for 4 days, as in previous studies [40], and these fish formed the positive-control group. A total of 30 other fish from the control group were exposed to clean water for 4 days, and these fish formed the negative-control group. Three replicate aquariums received the preparation in terms of their respective treatment groups. Each aquarium contained 10 fish. The conditions and process of the experiment were identical to those for the feeding experiment. Upon the cessation of CuSO_4_ stress, the FI in each treatment group was confirmed to verify the reliability of the experiment.

After the exposure to Cu ended, 5 fish in each aquarium were given an anesthetic in water with ethyl carbamate. After 5 min, the fish is taken out and kept on ice. Each treated fish undergoes the same anesthetic concentration and time. By means of caudal puncture, the researchers collected blood from the fish with a heparinized syringe. The blood was centrifuged for 5 min at 1000× *g* at 4 °C within 1 h of collection. The plasma and red blood cells were isolated in the blood in order to measure the hematochemical and hematological indices. The plans put forward by previous reports [41,42] helped with the specification of the red blood cell (RBC) count, hematocrit (Hct), and hemoglobin (HbC) concentrations in the blood. This study obtained the following variables: the mean corpuscular Hb concentration (MCHC), mean corpuscular volume (MCV), and mean corpuscular hemoglobin content (MCH), according to the means of Li et al. (2020) [41]. Red blood cells were stored at −80 °C for subsequent measurements of the Na^+^, K^+^-ATPase, glutamate-pyruvate transaminase (GPT), glutamate–oxaloacetate transaminase (GOT), and lactate dehydrogenase (LDH) activities. Gill samples were promptly removed and preserved at −80 °C for subsequent analysis. This step was crucial for measuring the superoxide anion (O_2_^·−^), hydroxyl radical (OH), reduced glutathione (GSH), and malondialdehyde (MDA) levels. Additionally, assessments of the CAT, GPx, SOD, Na^+^, K^+^-ATPase, GPT, GOT, and glutathione-S-transferase (GST) activities were conducted. The activities of caspase-8, caspase-3, and caspase-9 were also evaluated, providing insights into the apoptotic processes.

#### 2.2.4. Metabolic Experiments

Following the cessation of Cu exposure, the researchers selected and weighed five fish from the 0.0 FA (negative-control), 0.0 FA+Cu (positive-control), 0.1 FA+Cu, 0.2 FA+Cu, 0.3 FA+Cu, and 0.4 FA+Cu groups (weights denoted as W and expressed in grams) in order to conduct metabolic studies. The measurements of the oxygen consumption rate (OCR) (expressed in mg g^−1^ h^−1^) and ammonia excretion rate (AER) (expressed in mg g^−1^ h^−1^) were conducted in line with the methods suggested by Li et al. (2020) [35], albeit with minor modifications. For this purpose, a large aquarium was filled with 100 liters of fresh tap water, which was then brought to ambient room temperature (22 °C). To ensure a state of saturated dissolved oxygen (DO), the water was continuously aerated using an SB-748 aerator (Shanghai, China) for a 24 h period. The initial DO concentration (IDO) in the water (measured in mg L^−1^) was accurately determined using a Leici JPBJ-608 dissolved-oxygen analyzer (Shanghai, China). Detection kits (Jiancheng, Nanjing, China) were employed to determine the initial ammonia concentrations (IACs) (mg L^−1^) in water. Prior to the commencement of the measurements, a quantity of water saturated with dissolved oxygen (DO), amounting to 40 times the weight of the fish, was carefully transferred to a specialized necking bottle. This bottle, made of polyethylene terephthalate, featured a mouth diameter of 4.5 cm and held a total volume of 4.5 liters. Subsequently, five fish were gently placed inside the bottle. To remove any excess air trapped within, the bottle’s walls were methodically pressed. Immediately, the bottle mouth was closed, and the timing was started. The experiment lasted for 0.5 h, which was used as the duration time (DT) (h). At the experiment’s conclusion, immediate measurements were taken for both the final dissolved oxygen (FDO) and final ammonia concentration (FAC), each expressed in mg L^−1^. It was crucial to ensure that the FDO concentration did not fall below 5.0 mg L^−1^ throughout the experiment to prevent physiological stress in the subjects. The OCR and AER were calculated on a per hour and per gram of fish body mass basis. Each treatment was subjected to three independent experimental repetitions to ensure data reliability.

OCR = (IDO − FDO) × V/(0.5 × W)

AER = (FAC − IAC) × V/(0.5 × W)

O:N ratio = (14 × OCR)/(16 × AER)

The fish were sacrificed at the College of Life Sciences, Neijiang Normal University (Neijiang, Sichuan, China). The procedures detailed previously were approved by the Neijiang Normal University’s Institutional Animal Care and Use Committee (IACUC). These practices aligned with the ethical standards set by the Chinese Institute of Chemical Biology’s Institutional Ethics Committee.

### 2.3. Cell Experiment

#### 2.3.1. Apoptosis Induction

Using the technique proposed by Li et al. (2013) [43], with only slight modifications, carp red blood cell apoptosis was induced. The red blood cells were isolated from three carps in the control group. The dissolution of CuSO_4_·5H_2_O into the PCS assisted with the fresh preparation of the stock solution. The solution was diluted in PCS to the expected concentrations of the control, 1.0, 2.0, 3.0, 4.0, and 5.0 μM of Cu. The isolated red blood cells were subsequently resuspended in the solutions to produce final concentrations of 1% (*v*/*v*). There were 4 replicates for every concentration. After 9 h of incubation at 37 °C, the mentioned specimens received 5 min of centrifugation at 4 °C and 1000× *g*. Subsequently, the Hb concentration was measured by harvesting the supernatants and collecting red blood cells to determine the phosphatidylserine (PS) exposure. The hemolysis and apoptosis rates in the carp red blood cells were calculated in order to analyze their relationship with the Cu level.

#### 2.3.2. Cytoprotection Assays

In this study, the appropriate concentration of Cu to induce apoptosis was 4.0 μM, which was applied in the experiments described below. An increase in the Cu concentration failed to induce higher apoptosis levels but did induce lower ones. The cytoprotection experiment was implemented using the technique of Li et al. (2017) [44], with only slight modifications. The red blood cells were first isolated from the control, 0.0, 0.1, 0.2, 0.3, and 0.4 FA groups, followed by their suspension in the PCS at a hematocrit of 2%. CuSO_4_·5H_2_O was dissolved in PCS to obtain a Cu concentration of 8.0 μM. The CuSO_4_·5H_2_O solution was subsequently added to the red blood cell suspensions at 1:1 (*v*/*v*) to induce apoptosis, with the exception of the control group. The operation was repeated four times for every treatment. Following 9 h of incubation at 37 °C, the red blood cells were collected to calculate the hemolysis, PS exposure, O_2_^·−^, hydrogen peroxide (H_2_O_2_), methemoglobin (Met-Hb), MDA, cytochrome c, and GSH levels and the activities exhibited by caspase-9, CAT, caspase-8, GPx, caspase-3, and SOD.

#### 2.3.3. Hemolysis and Apoptosis Measurement

Using the technique of Li et al. (2020) [41], the hemolysis in the cultured carp red blood cells was quantified. The Annexin V-FITC Apoptosis Detection Kit (Beyotime, Nantong, China) was used to assess the PS exposure in the fish red blood cells, following previous methods [43]. Flow cytometry (Becton Dickinson, Heidelberg, Germany) was used to quantify the level of PS exposure in the fluorescence channel FL^−1^, characterized by an emission wavelength of 530 nm and an excitation wavelength of 488 nm.

#### 2.3.4. Cytochrome c Measurement

A commercial extraction kit (Beyotime, Nantong, China) was used to isolate the mitochondrial and cytosolic proteins in the fish red blood cells. An ELISA kit (ElabScience, Wuhan, China) was used to evaluate the cytochrome c released from the cytosol mitochondria, as previously explained [45]. The absorbance values of the specimens were measured using a microplate reader at 450 nm.

### 2.4. Biochemical Analysis

Following the report by Huang et al. (2019) [46], we measured the GPT and GOT activities. The methods described by Lin et al. (2011) [47] were employed to determine the concentrations of O_2_^·−^ and MDA. The assay procedures outlined by Chen et al. (2009) [48] were utilized to assess the activities of LDH and Na^+^, K^+^-ATPase. The SOD, CAT, and GPx active states were assayed via the approaches of Jiang et al. (2009) [49]. The H_2_O_2_, GSH, and Met-Hb levels in the red blood cells were determined according to Li et al. (2016) [50]. The measurements of the ·OH levels and GST activity were conducted using the methodologies proposed by Li et al. (2019) [51]. The Hb concentration was determined by employing the Drabkin (1946) technique [52]. Additionally, the protein content was quantified based on the method delineated in a previous study [53].

The indicators above were determined using detection kits (Nanjing Jiancheng Bioengineer Institute, Nanjing, China) according to their instructions. A microplate reader (Thermo, Waltham, MA, USA) was used to quantify the enzyme activity and substance content in the specimens in different wavelength states [40].

### 2.5. Caspase Measurement

Utilizing detection kits supplied by Beyotime (Nantong, China), the activities of caspase-8, caspase-3, and caspase-9 were quantified. This quantification involved the assessment of the specific cleavages of their respective substrates: Ac-IETD-pNA for caspase-8, Ac-DEVD-pNA for caspase-3, and Ac-LEHD-pNA for caspase-9. The cleavage process leads to the liberation of pNA, a technique previously described in [45]. A microplate reader (Thermo, Waltham, MA, USA) was used to quantify the caspase activity in the specimens at 405 nm.

### 2.6. Statistical Analysis

The normality of the data was verified using the Kolmogorov–Smirnov test. The data, which underwent a one-way analysis of variance (ANOVA) [38], are expressed as means ± standard deviations (SDs). Based on Duncan’s multiple-range assay [54], this study found noticeable differences in the exposure groups. Tukey’s multiple-range test was used to determine significant differences between the control and exposure groups. The level of significance adopted was 95% (α = 0.05). The statistical analysis was performed using SPSS 15.0 for Windows software (Chicago, USA; Version 15.0).

## 3. Results

### 3.1. Influences of Dietary FA on Growth Performance and Respiratory Metabolism in Cu-Treated Carp

Table 2 illustrates the impacts of the diets with varying FA levels on the growth parameters of the juvenile Jian carp. Compared with the control group, the inclusion of dietary FA significantly improved the FBW, WG, SGR, and FI (*p* < 0.05). Notably, no mortalities were recorded throughout the study. The optimal WG and SGR were observed in the fish that consumed the diet containing 0.30 g of FA per kilogram of diet, whereas the highest FBW and FI were achieved with the diet containing 0.40 g of FA per kilogram. Thus, the proper concentration of FA for growth is between 0.30 and 0.40 g kg^−1^ (Table 2).

In comparison with the control group, the final ammonia concentrations (FACs) were decreased and the final dissolved oxygen (FDO) was enhanced in the water of the Cu-treatment group (*p* < 0.05) (Table 3). In the Cu-treatment group, the inclusion of dietary FA resulted in a significant reversal of the previously mentioned parameters, as shown in Table 3 (*p* < 0.05). Additionally, as detailed in Table 3, the metabolic index revealed that both the OCR and AER decreased in the Cu-exposed carp, while the O:N ratio increased (*p* < 0.05). However, in the Cu-treatment group, dietary FA reversed this index, which gradually changed with increasing FA concentrations in the diet (*p* < 0.05) (Table 3).

### 3.2. Influences of Dietary FA on Biochemical Parameters in Gills of Cu-Treated Carp

**Figure 1:**
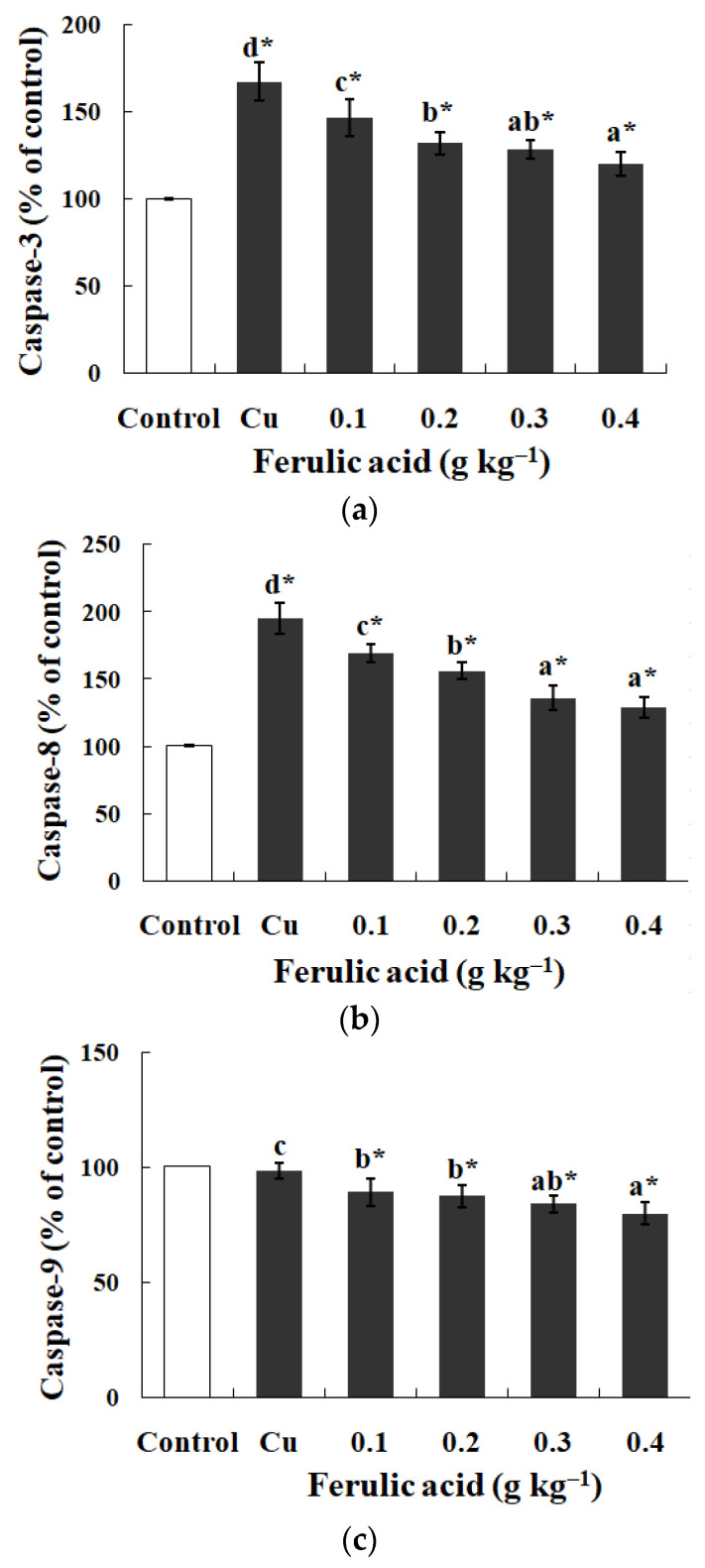
The data represent the means ± SDs of four replicates. The values that do not share superscript letters indicate significant differences among the exposure groups (*p <* 0.05). The order of the letters represents the values ranging from small to large. The significance between the control and exposure groups is indicated by * *p* < 0.05. (**a**) The activities of caspase-3 in the gills of the carp fed diets containing different levels of ferulic acid (FA) (grams per kg^−1^ of diet) for 30 days, followed by exposure to 12.5 μM of copper (Cu) for 4 days. The regression equations were Y = 250.0X^2^ − 213.0X + 166.4 (*R*^2^ = 0.989). (**b**) The activities of caspase-8 in the gills of the carp fed diets containing different levels of ferulic acid (FA) (grams per kg^−1^ of diet) for 30 days, followed by exposure to 12.5 μM of copper (Cu) for 4 days. The regression equations were Y = 221.4X^2^ − 253.5X + 194.4 (*R*^2^ = 0.991). (**c**) The activities of caspase-9 in the gills of the carp fed diets containing different levels of ferulic acid (FA) (grams per kg^−1^ of diet) for 30 days, followed by exposure to 12.5 μM of copper (Cu) for 4 days. The regression equations were Y = 59.71X^2^ − 65.66X + 97.43 (*R*^2^ = 0.956).

In comparison with the control group, the Na^+^, K^+^-ATPase, GPT, and GOT activities exhibited effectual decreases while the caspase-3 and -8 activities were apparently enhanced in the gills of the Cu-treatment group (*p* < 0.05) (Table 4 and Figure 1). However, dietary FA reversed the parameters above and reduced the caspase-9 activity in the Cu-treatment group (*p* < 0.05) (Table 4 and Figure 1). When the FA concentration was elevated to 0.30 g kg^−1^, the GOT activity in the gills of the Cu-treated carp peaked, while the caspase-8 activity reached its lowest level, as indicated in Table 4 (*p* < 0.05).

Relative to the control group, there was a notable increase in the O_2_^·−^, ·OH, and MDA levels and GPx activity in the Cu-treated carp, as shown in Table 4 (*p* < 0.05). In contrast, the SOD, CAT, and GST activities, along with the GSH levels, exhibited a downward trend (*p* < 0.05). However, the inclusion of dietary FA markedly reduced the O_2_^·−^, ·OH, and MDA levels in the gills of the Cu-treated carp, a trend that was inversely proportional to the FA concentrations in the diets (*p* < 0.05) (Table 4). Furthermore, dietary FA significantly elevated the GSH levels and CAT, SOD, GST, and GPx activities, with these increases corresponding to higher FA concentrations in the diets (*p* < 0.05) (Table 4).

### 3.3. Influences of Dietary FA on Hematology and Biochemical Index in Red Blood Cells of Cu-Treated Carp

Relative to those of the control, the blood parameters of the Cu-treated carp, such as the Hct, RBC, HbC, and MCH, were significantly diminished following the Cu treatment, as shown by the data in Table 5 (*p* < 0.05). However, the addition of FA to the diet counteracted the Cu-induced reductions in these metrics (*p* < 0.05) (Table 5). It was observed that the levels of HbC, RBC, Hct, and MCH in the blood of the Cu-treatment group progressively increased with the higher FA concentrations in their diets (*p* < 0.05) (Table 5).

In contrast to the control, the Na^+^, K^+^-ATPase, LDH, GOT, and GPT activities in the red blood cells of the Cu-treatment group showed marked decreases, as documented in Table 6 (*p* < 0.05). Yet, these decreases were reversed with the introduction of dietary FA in the Cu-exposed groups (*p* < 0.05) (Table 6). Most notably, an increase in FA to 0.30 g kg^−1^ in the diet led to the highest recorded GOT activity in the red blood cells of the Cu-treated carp, as indicated in Table 6 (*p* < 0.05).

### 3.4. PS Exposure (a Biomarker of Apoptosis) and Hemolysis Caused by Cu in Carp Red Blood Cells

**Figure 2:**
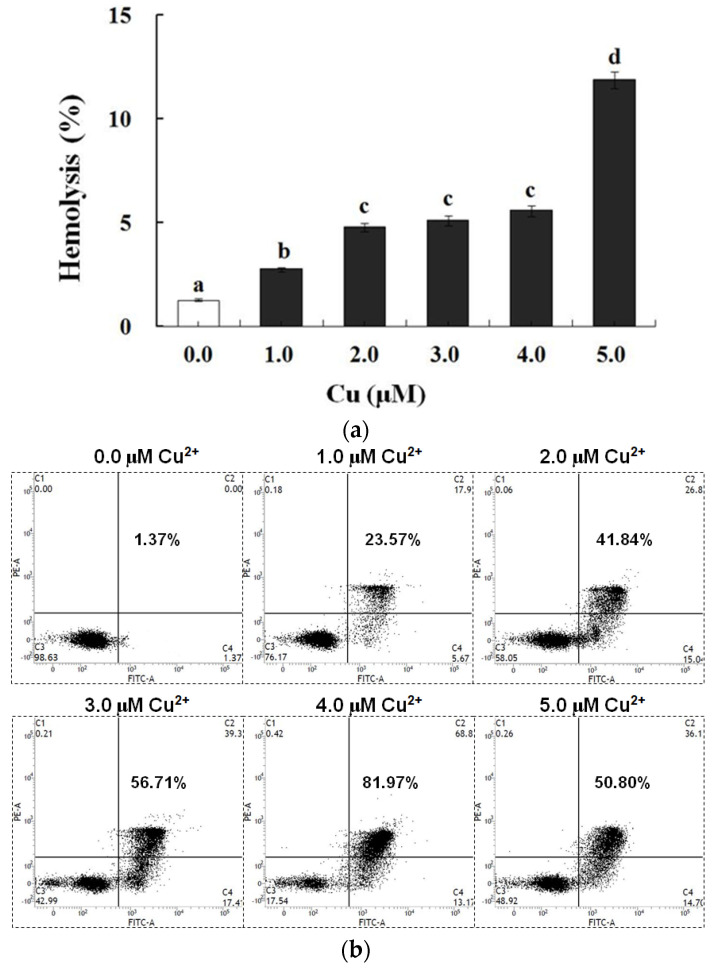

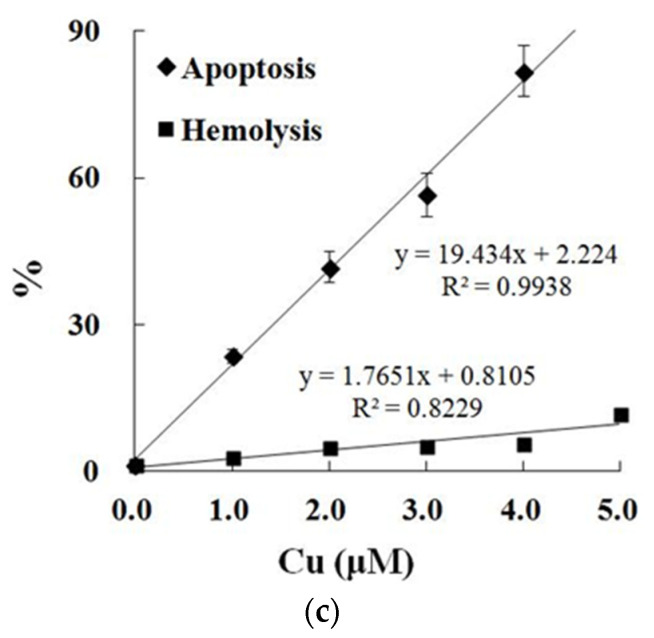
Cu-induced cellular model of hemolysis and apoptosis in carp red blood cells. (**a**) The levels of hemolysis in Cu-treated carp red blood cells. The data represent the means ± SDs of four replicates. The values that do not share a superscript are significantly different (*p* < 0.05). (**b**) The levels of annexin binding in Cu-treated carp red blood cells. This experiment was repeated three times with similar results achieved. (**c**) The relationship between apoptosis and hemolysis levels in carp red blood cells and Cu level in PCS. The data represent the means ± SDs of three replicates.

In contrast to the control, exposure to Cu increased the annexin binding level in a dose-dependent manner (0.0–4.0 μM), which showed the increase in the PS exposure of the carp red blood cells (*p* < 0.05) (Figure 2a,c). The level of hemolysis gradually increased as the Cu concentration increased, which illustrated the increase in the impairment in the carp red blood cells (*p* < 0.05) (Figure 2b,c). A further increase in the Cu concentration to 5.0 μM brought about lower PS exposure and higher hemolysis (*p* < 0.05) (Figure 2a,b). Therefore, the appropriate concentration of Cu to induce apoptosis was 4.0 μM, which was applied in all of the experiments.

### 3.5. Effects of Dietary FA on Apoptosis Parameters in Carp Red Blood Cells Treated with Cu

**Figure 3:**
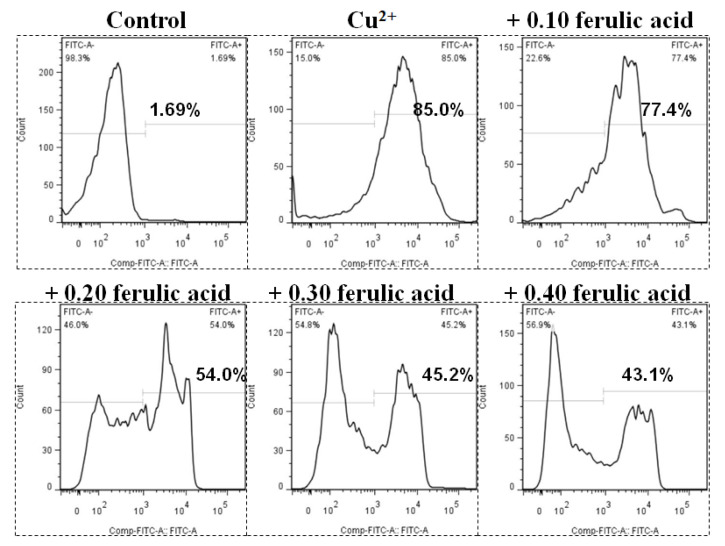
The levels of annexin V-FITC binding in red blood cells of carp fed diets containing different levels of ferulic acid (FA, g kg^−1^) for 30 days, followed by exposure to 4.0 μM copper (Cu) for 9 h in vitro. This experiment was repeated three times with similar results achieved. The regression equations were Y = 1.857X^2^ − 1.902X + 0.877 (*R*^2^ = 0.954). The optimal FA concentration was 0.512 g kg^−1^ diet.

**Figure 4:**
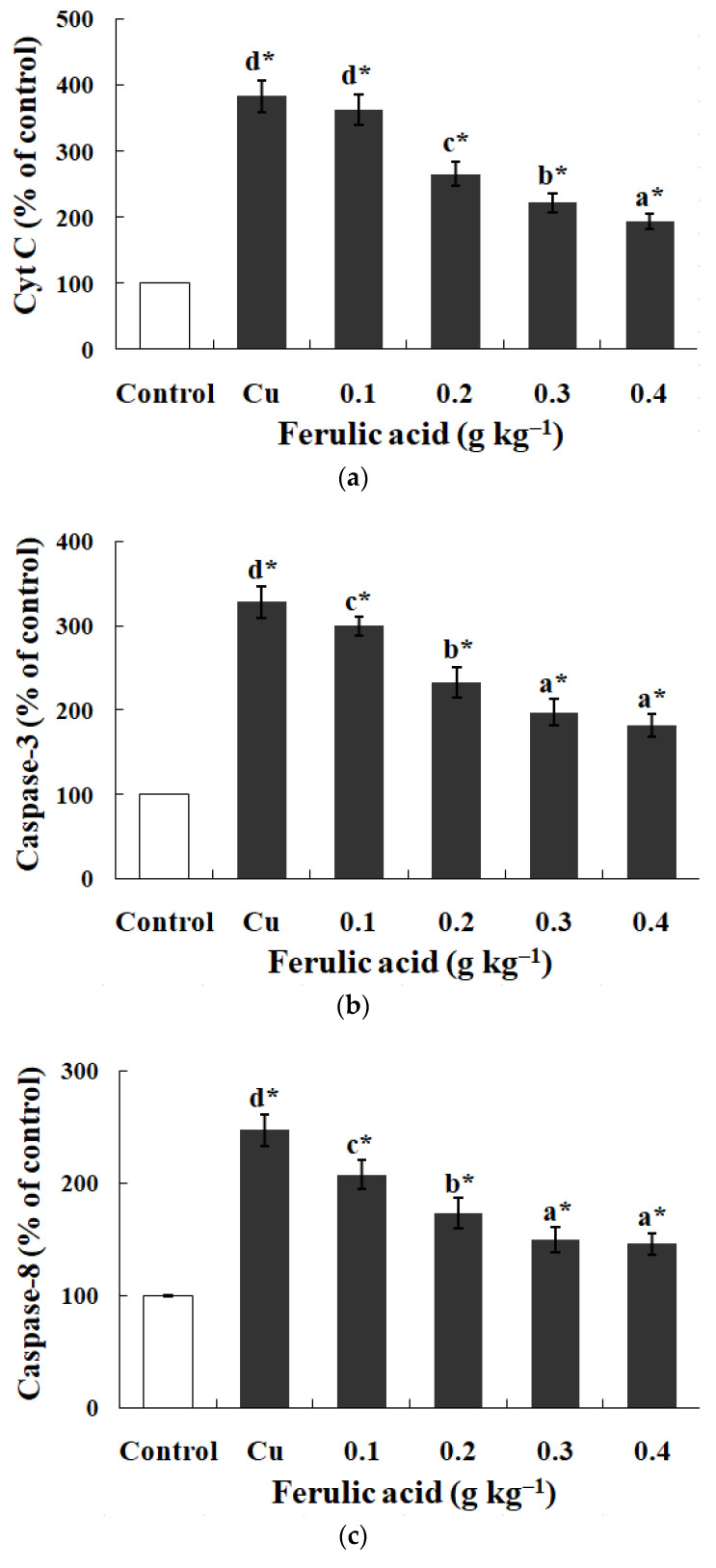

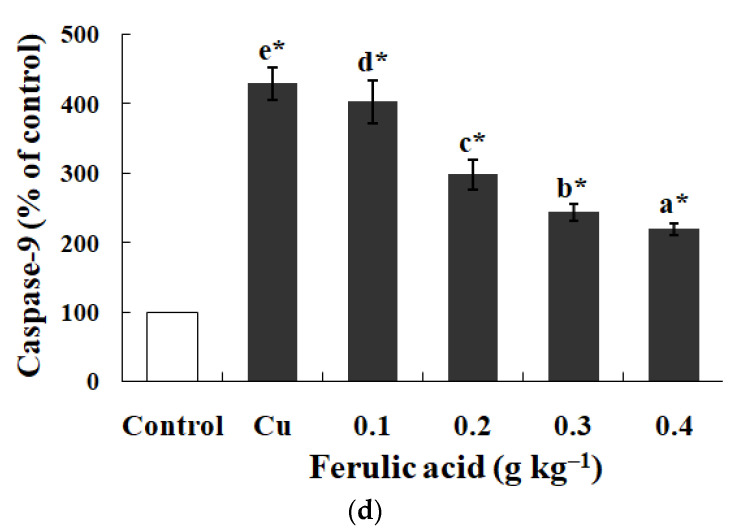
The data represent the means ± SDs of four replicates. The values that do not share superscript letters indicate significant differences among the exposure groups (*p* < 0.05). The order of the letters represents the values ranging from small to large. The significance between the control and exposure groups is indicated by * *p* < 0.05. (**a**) The levels of cytochrome c in red blood cells of carp fed diets containing different levels of ferulic acid (FA) (grams per kg^−1^ of diet) for 30 days, followed by exposure to 4.0 μM copper (Cu) for 9 h in vitro. The regression equations were Y = −522.0X + 389.4 (*R*^2^ = 0.951). (**b**) The activity of caspase-3 in red blood cells of carp fed diets containing different levels of ferulic acid (FA) (grams per kg^−1^ of diet) for 30 days, followed by exposure to 4.0 μM copper (Cu) for 9 h in vitro. The regression equations were Y = −395.0X + 327.0 (*R*^2^ = 0.958). (**c**) The activity of caspase-8 in red blood cells of carp fed diets containing different levels of ferulic acid (FA) (grams per kg^−1^ of diet) for 30 days, followed by exposure to 4.0 μM copper (Cu) for 9 h in vitro. The regression equations were Y = 578.5X^2^ − 490.4X + 248.1 (*R*^2^ = 0.997). (**d**) The activity of caspase-9 in red blood cells of carp fed diets containing different levels of ferulic acid (FA) (grams per kg^−1^ of diet) for 30 days, followed by exposure to 4.0 μM copper (Cu) for 9 h in vitro. The regression equations were Y = −579.0X + 434.4 (*R*^2^ = 0.951).

Relative to the control, the annexin binding, cytochrome c level, and caspase-8, caspase-3, and caspase-9 activities presented obvious increases in the carp red blood cells that were exposed to Cu (*p* < 0.05) (Figure 3 and Figure 4). However, these parameters presented a gradual downtrend as the concentration of dietary FA increased in the carp red blood cells exposed to Cu (*p* < 0.05) (Figure 3 and Figure 4). With the administration of FA at a concentration of 0.30 g per kg^−1^ of diet, the caspase-3 and caspase-8 activities were at their minimum values in the carp red blood cells exposed to Cu (*p* < 0.05) (Figure 3 and Figure 4).

### 3.6. Effects of FA on Oxidative Lesion Parameters in Carp Red Blood Cells Treated with Cu

**Figure 5:**
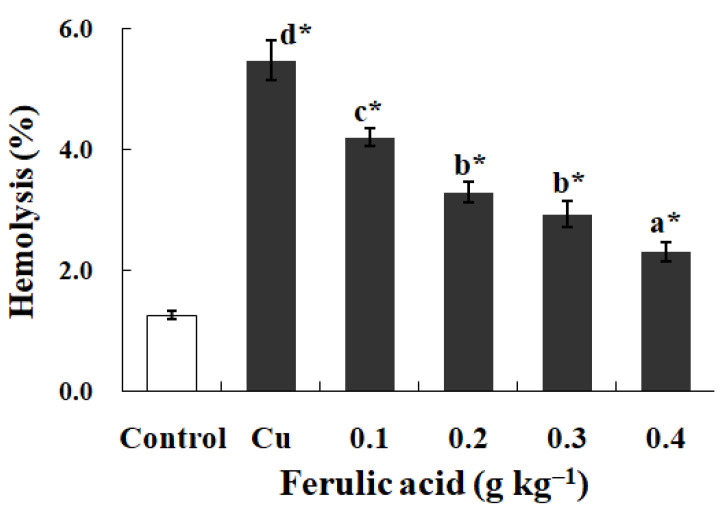
The levels of hemolysis in red blood cells of carp fed diets containing different levels of ferulic acid (FA) (grams per kg^−1^ of diet) for 30 days, followed by exposure to 4.0 μM copper (Cu) for 9 h in vitro. The data represent the means ± SDs of four replicates. The values that do not share superscript letters indicate significant differences among the exposure groups (*p* < 0.05). The order of the letters represents the values ranging from small to large. The significance between the control and exposure groups is indicated by * *p* < 0.05. The regression equation was Y = 13.28X^2^ − 12.93X + 5.42 (*R*^2^ = 0.991).

As opposed to the control, the exposure to Cu noticeably promoted the hemolysis and increased the levels of O_2_^·−^, Met-Hb, H_2_O_2_, and MDA, as well as the SOD activity, in the carp red blood cells (*p* < 0.05) (Figure 5 and Table 7). However, dietary FA effectively decreased the abovementioned parameters in the red blood cells administered Cu (*p* < 0.05) (Figure 5 and Table 7). The parameters gradually decreased with the increase in the FA concentrations in the diets (*p* < 0.05) (Figure 5 and Table 7). When the FA concentration was increased to 0.3 g per kg^−1^ of diet, the Met-Hb level reached the lowest value in the carp red blood cells administered Cu (*p* < 0.05) (Table 7).

Upon comparison with the control group, it was observed that the Cu exposure markedly reduced the levels of GSH and decreased the activities of the GPx and CAT enzymes in the carp red blood cells, as indicated by the statistical significance in Table 7 (*p* < 0.05). Even so, dietary FA effectively enhanced these parameters in the red blood cells administered Cu (*p* < 0.05) (Table 7). When the FA concentration was increased to 0.3 g per kg^−1^ of diet, the CAT activity reached the highest value in the carp red blood cells administered Cu (*p* < 0.05) (Table 7).

## 4. Discussion

Cu can inhibit both respiration and photosynthesis in algae. Thus, CuSO_4_ is a very effective algicide [55]. When used as an algicide, the concentration of CuSO_4_ is from 10 to 40 times higher than that of CuSO_4_ used as a protective agent for aquatic animals [2,5]. High doses of CuSO_4_ may be acutely toxic to fish, and they may cause problems in ponds with low dissolved oxygen concentrations immediately after treatment [55].

### 4.1. Dietary FA Improved Respiratory Metabolism in Cu-Treated Fish

According to the research, 30 days of dietary FA supplement administration significantly promoted carp growth. Consistent with observations in tilapia species, as documented in [56,57], the growth patterns appear to be intricately linked with the processes governing energy metabolism in aquatic species. In animal organisms, the fundamental physiological processes of respiration and excretion play pivotal roles in the regulation of energy metabolism. These activities are integral to the maintenance of vital functions and the overall energy balance [58]. The respiratory rate manifests in the OCR, which is the critical indicator of the metabolic and aerobic energy status in fish [35]. In fish, proteins predominantly serve as the primary source of energy, as indicated in [38]. In the case of teleost fish, the catabolic processing of these proteins primarily results in the production of ammonia, a key metabolic byproduct, as detailed in [40]. The O:N ratio serves as an informative indicator of the shifts in the utilization of energy substrates (nutrients) by animals when faced with different environmental stressors, as noted in [58]. A heightened O:N ratio is indicative of increased lipid and carbohydrate metabolism. In this study, dietary FA inhibited decreases in the OCR and AER and prevented the enhancement of the O:N ratio in the carp exposed to Cu. These results are in accordance with the finding that Cu exposure induced the significant inhibition of the OCR and AER and enhanced the O:N ratio in crayfish [58]. No reports have been published on the effects of FA on the abovementioned indicators in fish. These results confirm that the Cu exposure induced an inhibition of the respiratory metabolism in the fish. However, dietary FA improved the respiratory metabolism in the Cu-treated fish.

### 4.2. Dietary FA Suppressed Oxidative Lesions and Apoptosis in Gills of Cu-Treated Fish

In fish respiration, the primary roles of the gills are the removal of nitrogenous waste and the facilitation of gas exchange [7]. However, the gills are the primary target organ for the toxic action of Cu in fish [5]. Na^+^, K^+^-ATPase is pivotal in sustaining the ion electrochemical gradients across the fish gill plasma membrane, as cited in [59]. Any decline in the activity of Na^+^, K^+^-ATPase has the potential to impair both the respiratory efficiency and ion regulation within these organisms [5]. GPT and GOT are key enzymes in protein metabolism, and their activities are indicative of amino acid utilization in fish organs [20]. According to the research, dietary FA protected against the Cu-induced decrease in the Na^+^, K^+^-ATPase, GPT, and GOT activities in the gills of the carp. This study aligns with the finding that Cu exposure reduced the Na^+^, K^+^-ATPase activity in the gills of neotropical fish [5], but it is not in line with the finding that Cu induced an increase in the GPT and GOT activities in the hepatopancreases of Crucian carp [40]. Variations in organ sensitivity in fish might account for the observed differences. Fish gills, being the initial point of contact with environmental contaminants, exhibit a heightened susceptibility to toxic substances [11]. This increased vulnerability stems from their expansive surface area, which enhances the interaction and absorption of toxins, as well as from the less effective detoxification mechanism compared with that in the liver [11]. Data on the impact of FA on the GPT and GOT activities in fish organs are limited. Current findings suggest that Cu exposure results in a decline in some gill functions. Conversely, dietary FA seems to mitigate the impairment in the gill functions caused by Cu in fish.

In fish, the typical functions of the gills may be associated with their ROS levels. ROS varieties such as O_2_^·−^, H_2_O_2_, and ·OH play a role in oxidizing the cellular components, including lipids, proteins, and key enzymes. This oxidation process results in the formation of MDA and the inactivation of these enzymes [43]. In the current research, dietary FA inhibited the increase in the O_2_^·−^, ·OH, and MDA levels in the gills of carp treated with Cu, which aligns well with the finding that Cu caused the generation of ROS and cellular-component oxidation in fish gills [19], as well as with the finding that dietary FA decreased the MDA values in the liver of tilapia [57]. Studies indicate that Cu exposure leads to a decrease in certain gill functions and an increase in ROS production, which, in turn, causes the oxidation of the cellular components, highlighting Cu’s role in inducing oxidative damage in fish gills. However, dietary FA inhibits Cu-caused oxidative lesions in fish gills.

ROS can stimulate caspase-8 and -9 by activating the clustered receptors of death (such as CD95) and cytochrome c that are released, thereby activating caspase-3 and successively exposing PS in fish red blood cells [45]. The current study showed that the dietary FA significantly decreased the activities of caspase-8, -9, and -3 in the carp gills treated with Cu. This discovery is in good agreement with the finding that Cu stimulated the expression of caspase-8 in zebrafish gills [12] and increased the caspase-3 level in carp head kidney [60], as well as with the finding that intravenous FA administration abrogated the elevation of the cytochrome c release and cleaved the caspase-3 levels and apoptosis in the ischemic cortexes of rats [61]. These results confirm that dietary FA could quench apoptosis in the extrinsic pathway by inhibiting the elevation of the caspase-8 and -3 activities, as well as quench apoptosis in the intrinsic pathway by inhibiting the elevation of the caspase-9 and -3 activities or the release of cytochrome c in the gills of fish treated with Cu. However, the information regarding the effects of FA on cytochrome c in the gills of fish treated with Cu is not clear. Further research is needed to clarify this result.

Fish cells are equipped with antioxidant enzymes that are responsible for neutralizing intracellular reactive oxygen species (ROS) and preventing the oxidation of the cellular components, as referenced in [62,63]. GSH, which serves as a primary non-enzymatic antioxidant, plays a crucial role in directly scavenging intracellular ROS [64]. Additionally, GST facilitates the conjugation of lipid peroxide breakdown products using GSH as a substrate [40]. In this study, FA supplementation in the diet was found to augment the GSH levels and SOD, GPx, CAT, and GST activities in the gills of the Cu-treated carp. This observation aligns with Jiang et al. (2011)’s findings on Cu exposure in the gills of carp [19], and with those on FA in tilapia intestines [56] and the cortexes and hippocampi of rats [65]. There is little information on how FA affects the abovementioned parameters in the gills of fish. As revealed, dietary FA could quench Cu-caused oxidative lesions and apoptosis by elevating the enzymatic antioxidant activity and non-enzymatic antioxidant levels in fish gills. 

### 4.3. Cu Caused Apoptosis and Hemolysis in Red Blood Cells of Fish

Research has proven that Cu initiates apoptosis in chicken hepatocytes and mouse B cells [66,67]. Here, carp red blood cells were administered Cu at different concentrations (0.0–5.0 μM). PS exposure acts as one apoptosis biomarker [45]. As the research shows, the PS exposure demonstrated a gradual increase along with the enhancement in the Cu concentration in the culture medium, which showed the rising apoptosis levels in the carp red blood cells. This finding aligns with the conclusion that Cu triggers fish hepatocyte apoptosis [68]. The level of apoptosis reached 4.0 μM Cu. The increase in the concentration of Cu failed to induce higher apoptosis levels but did induce lower ones. Cu is capable of initiating membrane constituent oxidation in human and rat red blood cells, resulting in membrane integrity loss [69,70]. The hemolysis rate was taken into account to assess the red blood cell impairment degree [71]. Inside the carp red blood cells, the hemolysis increased with the increase in the Cu concentration, as reported in a study on trout red blood cells [72]. The hemolysis at 5.0 μM of Cu was noticeably one level greater than that at 4.0 μM, making 4.0 μM the best Cu concentration for apoptosis induction. According to the work performed in the lab, 9 h was the most appropriate ROS incubation period to trigger apoptosis in the fish red blood cells [43]. Hence, this study selected carp red blood cells that underwent 9 h of incubation with 4.0 μM of Cu as the apoptosis model for the subsequent experimental processes.

### 4.4. Dietary FA Suppressed Oxidative Lesions and Apoptosis in Fish Red Blood Cells Treated with Cu

The primary functions exhibited by red blood cells include the transportation of O_2_ and the mediation of CO_2_ generation in respiration [35,73]. In red blood cells, Hb is one type of oxygen carrier protein, and it can bind to O_2_ to synthesize oxygenated Hb for the release of O_2_ in animal tissues [41]. Accordingly, dietary FA recuperated the Cu-induced decreases in the HbC, RBC, Hct, and MCH in the blood of the carp. Such an outcome effectively complies with the finding that Cu decreased the HbC, RBC, and Hct in the blood of rainbow trout [74]. To date, little information is available on the effect of FA on the abovementioned parameters in fish. Given these outcomes, Cu could reduce O_2_ transportation function through a decrease in the Hb concentration and red blood cell count in fish blood. In contrast, dietary FA could inhibit function impairment in fish administered Cu.

There may be a close association between how Cu affects the Hb concentration and red blood cell count and apoptosis and lesions in the red blood cells of fish. Here, dietary FA suppressed PS exposure and hemolysis due to Cu in carp red blood cells, which shows the ability of FA to protect against Cu-induced apoptosis and lesions in fish red blood cells. This result further supports the finding that Cu causes hemocyte apoptosis in white shrimp [16] and hemolysis in human erythrocytes [70]. FA prohibits the apoptosis of PC12 cells caused by kainic acid [75]. PS exposure is likely to be positively related to the caspases in fish red blood cells under Cu treatment. As explained by Mandal et al. (2002 and 2005), oxidative stress can induce death receptors, which is followed by the activation of caspase-8 and -3, which triggers the loss of aminophospholipid translocase activity and externalizing PS in the red blood cells of humans (extrinsic pathway) [76,77]. Based on previous studies, cytochrome c release from mitochondria stimulates caspase-9 with successive caspase-3 in fish red blood cells (intrinsic pathway) [45]. In this study, due to Cu, the cytochrome c levels and caspase-3, -8, and -9 activities were enhanced in carp red blood cells, which is in accordance with the finding that Cu initiated apoptosis via the extrinsic pathway involved with death receptors and caspase-8 in zebrafish gills [12], as well as with the finding that Cu induced apoptosis through the mitochondria-mediated pathway in chicken hepatocytes [66]. Therefore, apoptosis seems to be initiated via two different pathways in fish red blood cells treated with Cu: in the first pathway, Cu triggers the death receptors, thereby activating caspase-8, resulting in caspase-3 activation with successive PS exposure; in the second pathway, Cu causes the release of cytochrome c with successive caspase-9 activation, which triggers caspase-3 with successive PS exposure in fish red blood cells. This hypothesis supports the report on the gills of zebrafish after Cu treatment [12]. Although caspase 8 plays an important role in the external apoptotic pathway, the effect of Cu on death receptors in fish red blood cells is not clear. Further research is needed to clarify this hypothesis. Currently, not much is known about how Cu influences the apoptotic pathway in fish red blood cells. However, dietary FA prevented increases in the cytochrome c levels and the caspase-3, -8, and -9 activities caused by Cu in carp red blood cells, which aligns with the finding that intravenous FA abrogated the elevation of caspase-3 levels, as well as cytochrome c release, in the ischemic cortexes in rats [61]. How FA affects the apoptotic pathway in fish cells has not been formally reported. As revealed by the abovementioned results, FA could quench apoptosis by inhibiting the elevation of the caspase activities and cytochrome c levels caused by Cu in fish red blood cells.

Na^+^, K^+^-ATPase is needed to maintain the cytoplasmic ionic milieu for stopping colloidal osmotic lysis in red blood cells [40,73]. LDH is a critical enzyme in anaerobic glycolysis, which helps to generate energy [78] that protects the Na^+^, K^+^-ATPase activity in red blood cells [73]. As found by Tiihonenk and Nikinmaa (1991), carp red blood cells can use amino acids as a source of energy [79]. GPT and GOT are capable of utilizing amino acids in the tricarboxylic acid cycle to generate energy based on deamination [80,81]. The present work proves that Cu suppresses the Na^+^, K^+^-ATPase, LDH, GOT, and GPT activities in carp red blood cells, which is consistent with the reports on Na^+^, K^+^-ATPase in neotropical fish gills [5], LDH in estuarine fish muscle [82], and GOT and GPT in carp gills. Obviously, dietary FA recuperated the indicators described above in the red blood cells of carp administered Cu. Little attention has been given to the effect of FA on the Na^+^, K^+^-ATPase, LDH, GOT, and GPT activities in fish organs. As the results mentioned above prove, Cu stimulated the impairment of the normal function in the fish red blood cells; however, the dietary FA limited the harm by reversing this process. The reason for these results might be related to the gene expression of metabolic enzymes in the fish red blood cells. Further research is needed to clarify this hypothesis.

The reason for the influence exerted by Cu on normal function may be an association with ROS in fish red blood cells. Oxygenated Hb is able to automatically receive continuous oxidization to synthesize O_2_^·−^ and Met-Hb, which fails to bind or transport O_2_ in red blood cells [83]. The O_2_^·−^ dismutation is conducive to producing H_2_O_2_, which is capable of reacting with heme Fe^2+^ to generate ·OH [73]. ROS can significantly induce apoptosis and hemolysis in fish red blood cells [43]. As found from the research, dietary FA lowered the O_2_^·−^, H_2_O_2_, and Met-Hb levels in carp red blood cells that were exposed to Cu. Such a result conforms well to the finding that Cu exposure increases the intracellular ROS levels in fish gill cells and hepatocytes [8,84], as well as the finding that FA reduced the ROS levels in ultraviolet A-irradiated human dermal fibroblasts [85]. We still know little about how Cu and FA impact Hb oxidation in fish red blood cells. According to the mentioned results, dietary FA is capable of limiting the generation of ROS and the oxidization of Hb in fish red blood cells exposed to Cu.

Fish red blood cells show a strong sensitivity to ROS because of the enrichment in unsaturated fatty acids in the cellular membranes [43]. Here, dietary FA inhibited the MDA level increase in carp red blood cells under Cu treatment. This finding conforms to a report that MDA formation was enhanced in the hearts, livers, spleens, and brains of rainbow trout exposed to Cu [74], as well as a finding that FA decreased the MDA levels in d-gal-induced mice [86]. This result confirms that Cu could cause lipids to oxidize in fish red blood cells. However, dietary FA could inhibit lipid oxidation in fish red blood cells administered Cu. ROS and lipid oxidation play key roles in the induction of apoptosis [45]. The abovementioned results confirm that Cu is capable of stimulating ROS synthesis and cellular-component oxidation, as well as function loss, demonstrating that Cu triggers oxidation-related lesions in fish red blood cells. Even so, dietary FA inhibited both Cu-induced oxidative lesions and apoptosis by quenching the ROS synthesis and cellular-component-oxidizing process in fish red blood cells.

Enzymes critical to the detoxification of ROS in organisms include CAT, GPx, and SOD [87]. SOD enables the enzymatic degradation of O_2_^·−^ to H_2_O_2_ [88]. CAT helps detoxify H_2_O_2_ to water and O_2_ [89]. GPx degrades lipid hydroperoxides with GSH as the substrate [39]. Here, dietary FA reversed the Cu-triggered changes in the abovementioned indexes in the carp red blood cells. The present results are in good harmony with the finding that exposure to Cu enhanced the SOD active state in chicken hepatocytes [66], as well as the finding that FA increased the expression of CAT in ultraviolet-A-induced human fibroblasts [85]. It has been suggested that FA can restore antioxidant enzyme activities in fish red blood cells administered Cu. There is a close association between the detoxification of ROS and GSH level in the fish red blood cells administered Cu. In red blood cells, GSH serves as a main non-enzyme-related antioxidant and significant sulfhydryl group in enzymes and Hb [73]. According to the finding here, dietary FA helped to increase the GSH levels inside the carp red blood cells administered Cu. This conforms well to the finding that exposure to Cu lowered the GSH content in chicken hepatocytes [66], as well as the finding that FA treatment increased the GSH content in the brains of D-gal-treated mice [86]. The abovementioned results demonstrate that dietary FA is capable of quenching oxidative lesions and apoptosis triggered by Cu by enhancing the enzyme-related antioxidant activity and non-enzyme-related antioxidant levels in fish red blood cells. For this reason, FA can limit ROS production and cellular-component oxidation, as well as preserving the enzyme-related antioxidant activity and non-enzyme-related antioxidant levels, thereby protecting fish red blood cells from Cu-triggered oxidative lesions and apoptosis.

## 5. Conclusions

In summary, dietary FA improved respiratory metabolism in Cu-treated fish by suppressing Cu-induced oxidative lesions and apoptosis in the gills and red blood cells. Dietary FA limited Cu-induced oxidative lesions by upregulating the enzyme-related antioxidant activity and non-enzyme-related antioxidant levels, depressing the production of ROS and the oxidation of the cellular components, and restoring the metabolic enzyme activities in the gills and red blood cells of the fish. Moreover, dietary FA quenched Cu-induced apoptosis by inhibiting ROS production and cellular-component oxidation and reducing the caspase activities and cytochrome c levels in the gills and red blood cells of the fish. Thus, FA can be used to suppress Cu stress in fish. 

## Figures and Tables

**Table 1:** Composition and proximate analysis of the basal and ferulic acid (FA) diets.

Ingredients (%)	0.00 g kg^−1^	0.10 g kg^−1^	0.20 g kg^−1^	0.30 g kg^−1^	0.40 g kg^−1^
Fish meal	25.00	25.00	25.00	25.00	25.00
Soybean meal	30.55	30.55	30.55	30.55	30.55
Wheat flour	36.17	36.17	36.17	36.17	36.17
DL-methionine	0.42	0.42	0.42	0.42	0.42
Threonine	0.40	0.40	0.40	0.40	0.40
Fish oil	1.16	1.16	1.16	1.16	1.16
Soybean oil	1.80	1.80	1.80	1.80	1.80
Ca(H_2_PO_4_)_2_·H_2_O	1.50	1.50	1.50	1.50	1.50
Vitamin mixture ^1^	1.00	1.00	1.00	1.00	1.00
Mineral mixture ^2^	1.00	1.00	1.00	1.00	1.00
Microcrystalline cellulose	1.00	0.99	0.98	0.97	0.96
FA	0.00	0.01	0.02	0.03	0.04
Proximate analysis (%)					
Dry matter	93.78	93.67	93.70	94.10	93.84
Crude protein	33.90	33.82	34.06	34.03	33.91
Crude lipid	5.55	5.48	5.54	5.61	5.53
Crude ash	7.56	7.72	7.69	7.58	7.75

^1^ Provided the following per kg of mineral mix: FeSO_4_·7H_2_O (20% Fe), 69.70 g; CuSO_4_·5H_2_O (25% Cu), 1.20 g; ZnSO_4_·7H_2_O (23% Zn), 21.64 g; MnSO_4_·H_2_O (32% Mn), 4.09 g; Na_2_SeO_3_·5H_2_O (1% Se), 2.50 g; KI (4% I), 2.90 g; CaCO_3_, 897.98 g. ^2^ Provided the following per kg of vitamin mix: retinyl acetate (500,000 IU g^−1^), 0.80 g; cholecalciferol (500,000 IU g^−1^), 0.48 g; DL-α-tocopherol acetate (50%), 20.00 g; menadione (23%), 0.43 g; thiamin nitrate (90%), 0.11 g; riboflavine (80%), 0.63 g; pyridoxine HCl (81%), 0.92 g; cyanocobalamin (1%), 0.10 g; ascorhyl acetate (93%), 7.16 g; D-calcium pantothenate (90%), 2.73 g; niacin (99%), 2.82 g; D-biotin (2%), 5.00 g; meso-inositol (99%), 52.33 g; folic acid (96%), 0.52 g.

**Table 2:** Initial body weight (IBW), final body weight (FBW), weight gain (WG), specific growth rate (SGR), feed intake (FI), and feed efficiency (FE) of juvenile Jian carp fed diets containing graded levels of ferulic acid (FA) (grams per kg^−1^ of diet) for 30 days.

Item	0.00 FA	0.10 FA	0.20 FA	0.30 FA	0.40 FA	Regression Equation	*R* ^2^
IBW (g fish^−1^)	9.65 ± 0.31 ^a^	9.71 ± 0.36 ^a^	9.71 ± 0.33 ^a^	9.61 ± 0.30 ^a^	9.69 ± 0.32 ^a^	-	-
FBW (g fish^−1^)	19.48 ± 0.62 ^a^	20.45 ± 1.05 ^ab^	21.26 ± 0.71 ^bc^	22.49 ± 1.19 ^cd^	23.11 ± 1.08 ^d^	Y = 9.3X + 19.49	0.992
WG (g fish^−1^)	9.83 ± 0.59 ^a^	10.74 ± 0.73 ^ab^	11.55 ± 0.94 ^b^	12.88 ± 1.01 ^c^	13.43 ± 0.98 ^c^	Y = 9.34X + 9.82	0.988
SGR (% day^−1^)	2.34 ± 0.12 ^a^	2.48 ± 0.08 ^ab^	2.61 ± 0.20 ^b^	2.83 ± 0.13 ^c^	2.90 ± 0.15 ^c^	Y = 1.47X + 2.34	0.982
FI (g fish^−1^)	16.71 ± 0.37 ^a^	17.11 ± 0.46 ^a^	18.50 ± 0.28 ^b^	20.25 ± 0.26 ^c^	21.35 ± 0.43 ^d^	Y = 12.42X + 16.30	0.969
FE (%)	58.77 ± 2.70 ^a^	62.72 ± 3.00 ^a^	62.44 ± 5.00 ^a^	63.53 ± 4.37 ^a^	62.84 ± 3.73 ^a^	Y = −56.50X^2^ + 31.55X + 59.14	0.880

Values are means ± SDs of 4 replicates, with 20 fish in each replicate. The values that do not share superscript letters indicate significant differences within the same lines among the exposure groups (*p* < 0.05). The order of the letters represents the values ranging from small to large.

**Table 3:** Fish body weight (W), water volume (V), final dissolved oxygen (FDO), and final ammonia concentrations (FAC) in water and oxygen consumption rate (OCR), ammonia excretion rate (AER), and O:N ratio in juvenile Jian carp fed diet containing graded levels of ferulic acid (FA) (grams per kg^−1^ of diet) for 30 days, followed by exposure to 12.5 μM copper (Cu) for 4 days.

Item	0.00 FA	0.00 FA+Cu	0.10 FA+Cu	0.20 FA+Cu	0.30 FA+Cu	0.40 FA+Cu	Regression Equation	*R* ^2^
W (g fish^−1^)	21.30 ± 0.26	21.20 ± 0.25 ^a^	21.43 ± 0.38 ^a^	21.33 ± 0.35 ^a^	21.50 ± 0.30 ^a^	21.57 ± 0.29 ^a^	-	-
V (L bottle^−1^)	4.26 ± 0.05	4.24 ± 0.05 ^a^	4.29 ± 0.08 ^a^	4.27 ± 0.07 ^a^	4.30 ± 0.06 ^a^	4.31 ± 0.06 ^a^	-	-
FDO (mg L^−1^)	5.33 ± 0.09	6.39 ± 0.09 ^d^*	6.25 ± 0.13 ^cd^*	6.12 ± 0.10 ^c^*	5.89 ± 0.10 ^b^*	5.65 ± 0.13 ^a^*	Y = −2.14X^2^ − 0.98X + 6.39	0.998
FAC (μmol L^−1^)	71.51 ± 1.63	61.70 ± 0.96 ^a^*	62.96 ± 1.16 ^ab^*	64.15 ± 0.81 ^bc^*	66.04 ± 1.28 ^c^*	68.79 ± 1.11 ^d^*	Y = 26.00X^2^ + 6.82X + 61.81	0.996
OCR (mg g^−1^ h^−1^)	0.24 ± 0.01	0.16 ± 0.01 ^a^*	0.17 ± 0.01 ^ab^*	0.18 ± 0.01 ^b^*	0.20 ± 0.01 ^c^*	0.22 ± 0.01 ^d^*	Y = 0.214X^2^ + 0.064X + 0.16	0.997
AER (mg g^−1^ h^−1^)	0.030 ± 0.002	0.017 ± 0.001 ^a^*	0.019 ± 0.002 ^ab^*	0.020 ± 0.001 ^ab^*	0.023 ± 0.002 ^b^*	0.027 ± 0.002 ^c^*	Y = 0.035X^2^ + 0.009X + 0.017	0.997
O:N ratio	6.99 ± 0.37	8.06 ± 0.29 ^b^*	7.85 ± 0.45 ^ab^*	7.67 ± 0.58 ^ab^*	7.55 ± 0.68 ^ab^*	7.09 ± 0.51 ^a^	Y = −3.142X^2^ − 0.982X + 8.029	0.969

Initial dissolved oxygen (IDO) = 8.34 ± 0.22 mg L^−1^; initial ammonia concentrations (IAC) = 49.25 ± 2.53 μmol L^−1^; durative time (DT) = 0.50 h. Values are means ± SDs of 3 replicates, with 5 fish in each replicate. The values that do not share superscript letters indicate significant differences within the same lines among the exposure groups (*p <* 0.05). The order of the letters represents the values ranging from small to large. The significance between the control and exposure groups is indicated by * *p* < 0.05.

**Table 4:** The superoxide anion (O_2_^·−^), hydroxyl radical (·OH), malondialdehyde (MDA), and reduced glutathione (GSH) levels and Na^+^, K^+^-ATPase, glutamate-oxaloacetate transaminase (GOT), glutamate-pyruvate transaminase (GPT), superoxide dismutase (SOD), catalase (CAT), glutathione peroxidase (GPx), and glutathione-S-transferase (GST) activities in the gills of carp fed diets containing different levels of ferulic acid (FA) (grams per kg^−1^ of diet) for 30 days, followed by exposure to 12.5 μM of copper (Cu) for 4 days.

Item	0.00 FA	0.00 FA+Cu	0.10 FA+Cu	0.20 FA+Cu	0.30 FA+Cu	0.40 FA+Cu	Regression Equation	*R* ^2^
Na^+^, K^+^-ATPase (U mg^−1^ protein)	6.61 ± 0.45	4.09 ± 0.14 ^a^*	4.83 ± 0.25 ^b^*	5.52 ± 0.29 ^c^*	5.63 ± 0.31 ^c^*	6.07 ± 0.38 ^d^*	Y = −8.43X^2^ + 8.13X + 4.11	0.981
GOT (U g^−1^ protein)	440.10 ± 26.01	266.94 ± 13.46 ^a^*	320.54 ± 15.12 ^b^*	320.74 ± 24.03 ^b^*	354.36 ± 18.91 ^c^*	376.31 ± 13.12 ^c^*	Y = −221.40X^2^ + 339.50X + 273.10	0.936
GPT (U g^−1^ protein)	146.52 ± 8.72	79.24 ± 3.37 ^a^*	89.70 ± 5.10 ^b^*	104.07 ± 3.71 ^c^*	104.18 ± 5.27 ^c^*	113.08 ± 4.38 ^d^*	Y = −124.10X^2^ + 131.80X + 79.13	0.963
O_2_^·−^ (U g^−1^ protein)	23.43 ± 1.63	32.92 ± 1.99 ^c^*	29.72 ± 1.41 ^b^*	27.58 ± 1.63 ^ab^*	28.11 ± 1.61 ^ab^*	26.38 ± 1.66 ^a^*	Y = 40.07X^2^ − 30.71X + 32.68	0.932
·OH (U mg^−1^ protein)	17.78 ± 0.68	22.47 ± 1.13 ^c^*	20.28 ± 1.26 ^b^*	18.69 ± 0.83 ^ab^	18.98 ± 1.00 ^ab^	17.51 ± 1.08 ^a^	Y = 23.71X^2^ − 20.70X + 22.30	0.937
MDA (nmol mg^−1^ protein)	4.87 ± 0.25	6.81 ± 0.24 ^c^*	6.03 ± 0.30 ^b^*	5.99 ± 0.38 ^b^*	5.47 ± 0.34 ^a^	5.28 ± 0.31 ^a^	Y = 5.00X^2^ − 5.62X + 6.74	0.946
GSH (mg g^−1^ protein)	10.47 ± 0.50	8.18 ± 0.27 ^a^*	8.32 ± 0.37 ^a^*	9.12 ± 0.53 ^b^*	9.61 ± 0.50 ^b^	10.59 ± 0.74 ^c^	Y = 9.79X^2^ + 2.20X + 8.14	0.987
SOD (U mg^−1^ protein)	12.07 ± 0.75	9.87 ± 0.45 ^a^*	10.69 ± 0.52 ^ab^	10.62 ± 0.58 ^ab^	11.72 ± 0.87 ^bc^	12.87 ± 0.95 ^c^	Y = 13.07X^2^ + 1.80X + 10.00	0.957
CAT (U mg^−1^ protein)	4.44 ± 0.30	2.79 ± 0.20 ^a^*	3.49 ± 0.18 ^b^*	4.11 ± 0.23 ^c^	4.82 ± 0.26 ^c^	5.53 ± 0.31 ^c^*	Y = 6.81X + 2.79	0.999
GPx (U mg^−1^ protein)	100.73 ± 7.26	119.20 ± 5.85 ^a^*	126.90 ± 7.05 ^ab^*	126.28 ± 7.20 ^ab^*	134.12 ± 8.48 ^b^*	138.98 ± 6.87 ^b^*	Y = 47.00X + 119.60	0.928
GST (U mg^−1^ protein)	48.05 ± 2.37	33.14 ± 2.36 ^a^*	42.63 ± 1.39 ^b^	47.45 ± 2.85 ^bc^	46.62 ± 3.55 ^bc^	51.71 ± 3.44 ^c^*	Y = −103.20X^2^ + 82.41X + 34.02	0.931

The data represent the means ± SDs of four replicates. The values that do not share superscript letters indicate significant differences within the same lines among the exposure groups (*p* < 0.05). The order of the letters represents the values ranging from small to large. The significance between the control and exposure groups is indicated by * *p* < 0.05.

**Table 5:** The haematocrit (Hct), red blood cell count (RBC), haemoglobin concentration (HbC), mean corpuscular volume (MCV), mean corpuscular haemoglobin content (MCH), and mean corpuscular haemoglobin concentration (MCHC) in blood of carp fed diets containing different levels of ferulic acid (FA) (grams per kg^−1^ of diet) for 30 days, followed by exposure to 12.5 μM copper (Cu) for 4 days.

Item	0.00 FA	0.00 FA+Cu	0.10 FA+Cu	0.20 FA+Cu	0.30 FA+Cu	0.40 FA+Cu	Regression Equation	*R* ^2^
Hct (%)	0.48 ± 0.02	0.42 ± 0.02 ^a^*	0.43 ± 0.02 ^a^*	0.45 ± 0.02 ^ab^	0.46 ± 0.02 ^ab^	0.49 ± 0.03 ^b^	Y = 0.214X^2^ + 0.084X + 0.420	0.984
RBC (10^12^ L^−1^)	2.44 ± 0.11	2.24 ± 0.13 ^a^	2.25 ± 0.12 ^a^	2.32 ± 0.14 ^ab^	2.38 ± 0.15 ^ab^	2.49 ± 0.13 ^b^	Y = 1.36X^2^ + 0.087X + 2.24	0.994
HbC (g L^−1^)	100.58 ± 2.40	82.66 ± 4.54 ^a^*	85.95 ± 3.46 ^a^*	89.40 ± 3.85 ^ab^*	94.37 ± 4.43 ^bc^	99.22 ± 5.04 ^c^	Y = 32.28X^2^ + 27.76X + 82.72	0.999
MCV (fL cell^−1^)	197.31 ± 8.35	186.50 ± 10.59 ^a^	190.76 ± 11.60 ^a^	193.75 ± 11.64 ^a^	194.96 ± 11.90 ^a^	196.50 ± 10.54 ^a^	Y = −64.28X^2^ + 50.71X + 186.20	0.989
MCH (pg cell^−1^)	41.23 ± 2.41	36.93 ± 1.50 ^a^*	38.21 ± 1.36 ^ab^	38.57 ± 1.50 ^ab^	39.76 ± 2.66 ^b^	39.91 ± 1.27 ^b^	Y = −10.21X^2^ + 11.59X + 36.97	0.968
MCHC (g L^−1^)	208.85 ± 4.98	198.37 ± 10.90 ^a^	200.58 ± 8.07 ^a^	199.39 ± 8.58 ^a^	204.07 ± 9.58 ^a^	203.43 ± 10.33 ^a^	Y = −14.28X^2^ + 19.71X + 197.90	0.828

Values are means ± SDs (*n* = 4). The values that do not share superscript letters indicate significant differences within the same lines among the exposure groups (*p* < 0.05). The order of the letters represents the values ranging from small to large. The significance between the control and exposure groups is indicated by * *p* < 0.05.

**Table 6:** Lactate dehydrogenase (LDH), glutamate–oxaloacetate transaminase (GOT), glutamate–pyruvate transaminase (GPT), and Na^+^, K^+^-ATPase in red blood cells of carp fed diets containing different levels of ferulic acid (FA) (grams per kg^−1^ of diet) for 30 days, followed by exposure to 12.5 μM copper (Cu) for 4 days.

Item	0.00 FA	0.00 FA+Cu	0.10 FA+Cu	0.20 FA+Cu	0.30 FA+Cu	0.40 FA+Cu	Regression Equation	*R* ^2^
LDH (U g^−1^ hemoglobin)	118.20 ± 8.12	95.95 ± 6.14 ^a^*	109.78 ± 6.16 ^b^	108.52 ± 7.32 ^b^	111.50 ± 8.60 ^b^	127.19 ± 8.87 ^c^	Y = 64.20X + 97.74	0.831
GOT (U g^−1^ hemoglobin)	21.13 ± 1.32	16.45 ± 1.07 ^a^*	19.27 ± 1.10 ^b^	21.32 ± 1.60 ^bc^	22.87 ± 1.48 ^c^	22.12 ± 1.74 ^c^	Y = −54.57X^2^ + 36.76X + 16.32	0.989
GPT (U g^−1^ hemoglobin)	15.59 ± 1.04	12.57 ± 0.78 ^a^*	15.03 ± 0.90 ^b^	15.60 ± 1.11 ^b^	16.42 ± 1.02 ^b^	15.91 ± 1.00 ^b^	Y = −40.64X^2^ + 24.32X + 12.67	0.974
Na^+^, K^+^-ATPase (U mg^−1^ hemoglobin)	1.52 ± 0.05	1.14 ± 0.07 ^a^*	1.36 ± 0.06 ^b^	1.39 ± 0.07 ^b^	1.51 ± 0.10 ^b^	1.67 ± 0.11 ^b^	Y = 1.21X + 1.17	0.954

Values are means ± SDs (*n* = 4). The values that do not share superscript letters indicate significant differences within the same lines among the exposure groups (*p* < 0.05). The order of the letters represents the values ranging from small to large. The significance between the control and exposure groups is indicated by * *p* < 0.05.

**Table 7:** The levels of superoxide anion (O_2_^·−^), hydrogen peroxide (H_2_O_2_), met-hemoglobin (Met-Hb), malondialdehyde (MDA), and reduced glutathione (GSH) and the activities of superoxide dismutase (SOD), catalase (CAT), and glutathione peroxidase (GPx) in red blood cells of carp fed diets containing different levels of ferulic acid (FA) (grams per kg^−1^ of diet) for 30 days, followed by exposure to 4.0 μM copper (Cu) for 9 h in vitro.

Item	0.00 FA	0.00 FA+Cu	0.10 FA+Cu	0.20 FA+Cu	0.30 FA+Cu	0.40 FA+Cu	Regression Equation	*R* ^2^
O_2_^·−^ (U g^−1^ protein)	26.18 ± 1.87	35.61 ± 2.24 ^c^*	34.58 ± 1.82 ^bc^*	32.56 ± 2.22 ^b^*	31.62 ± 1.20 ^ab^*	29.48 ± 2.16 ^a^*	Y = −15.22X + 35.81	0.984
H_2_O_2_ (μmol g^−1^ protein)	40.43 ± 1.91	57.24 ± 3.82 ^c^*	56.36 ± 3.74 ^c^*	53.53 ± 2.64 ^bc^*	50.11 ± 2.56 ^ab^*	47.88 ± 3.03 ^a^*	Y = −24.97X + 58.01	0.972
Met-Hb (g L^−1^)	1.74 ± 0.10	2.02 ± 0.14 ^b^*	1.89 ± 0.09 ^b^	1.88 ± 0.07 ^b^	1.69 ± 0.08 ^a^	1.61 ± 0.10 ^a^	Y = −1.02X + 2.02	0.950
MDA (nmol mg^−1^ protein)	1.64 ± 0.09	2.22 ± 0.10 ^c^*	2.10 ± 0.15 ^bc^*	2.04 ± 0.14 ^bc^*	2.00 ± 0.12 ^ab^*	1.86 ± 0.11 ^a^*	Y = −0.82X + 2.21	0.961
GSH (μmol g^−1^ protein)	5.07 ± 0.31	3.63 ± 0.27 ^a^*	4.03 ± 0.16 ^b^*	4.32 ± 0.16 ^b^*	5.00 ± 0.19 ^c^	5.53 ± 0.31 ^d^	Y = 4.77X + 3.55	0.980
SOD (U mg^−1^ protein)	68.95 ± 5.46	81.91 ± 6.29 ^b^*	79.07 ± 4.99 ^ab^	77.05 ± 3.51 ^ab^	73.24 ± 5.01 ^a^	72.20 ± 4.86 ^a^	Y = −25.25X + 81.74	0.979
CAT (U mg^−1^ protein)	6.03 ± 0.33	4.10 ± 0.34 ^a^*	4.33 ± 0.26 ^a^*	4.89 ± 0.34 ^b^*	5.58 ± 0.32 ^c^	5.87 ± 0.44 ^c^	Y = 4.79X + 4.00	0.974
GPx (U mg^−1^ protein)	66.85 ± 5.14	45.38 ± 2.27 ^a^*	48.49 ± 3.71 ^ab^*	52.64 ± 3.50 ^b^*	58.56 ± 3.53 ^c^*	62.90 ± 4.01 ^c^	Y = 30.21X^2^ + 33.02X + 45.17	0.995

Values are means ± SDs (*n* = 4). The values that do not share superscript letters indicate significant differences within the same lines among the exposure groups (*p* < 0.05). The order of the letters represents the values ranging from small to large. The significance between the control and exposure groups is indicated by * *p* < 0.05.

## Data Availability

The data used to generate the results in this manuscript can be made available if requested from the corresponding author.
